# *CYP2C19* Genotype Prevalence and Association With Recurrent Myocardial Infarction in British–South Asians Treated With Clopidogrel

**DOI:** 10.1016/j.jacadv.2023.100573

**Published:** 2023-09

**Authors:** Emma F. Magavern, Benjamin Jacobs, Helen Warren, Gherardo Finocchiaro, Sarah Finer, David A. van Heel, Genes & Health Research Team, Damian Smedley, Mark J. Caulfield

**Affiliations:** a William Harvey Research Institute, Queen Mary University of London, London, United Kingdom; b The Wolfson Institute of Population Health, Queen Mary University of London, London, United Kingdom; c Cardiovascular Sciences Research Centre, St George's, University of London, London, United Kingdom; d The Blizard Institute, Queen Mary University of London, London, United Kingdom

**Keywords:** ischemic heart disease, pharmacogenomics, pharmacotherapy, preventive cardiology

## Abstract

**Background:**

Cytochrome P450 family 2 subfamily C member 19 (CYP2C19) is a hepatic enzyme involved in the metabolism of clopidogrel from a prodrug to its active metabolite. Prior studies of genetic polymorphisms in *CYP2C19* and their relationship with clinical efficacy have not included South Asian populations.

**Objectives:**

The objective of this study was to assess prevalence of common *CYP2C19* genotype polymorphisms in a British–South Asian population and correlate these with recurrent myocardial infarction risk in participants prescribed clopidogrel.

**Methods:**

The Genes & Health cohort of British Bangladeshi and Pakistani ancestry participants were studied. *CYP2C19* diplotypes were assessed using array data. Multivariable logistic regression was used to test for association between genetically inferred CYP2C19 metabolizer status and recurrent myocardial infarction, controlling for known cardiovascular disease risk factors, percutaneous coronary intervention, age, sex, and population stratification.

**Results:**

Genes & Health cohort participants (N = 44,396) have a high prevalence (57%) of intermediate or poor CYP2C19 metabolizers, with at least 1 loss-of-function *CYP2C19* allele. The prevalence of poor metabolizers carrying 2 *CYP2C19* loss-of-function alleles is 13%, which is higher than that in previously studied European (2.4%) and Central/South Asian populations (8.2%). Sixty-nine percent of the cohort who were diagnosed with an acute myocardial infarction were prescribed clopidogrel. Poor metabolizers were significantly more likely to have a recurrent myocardial infarction (OR: 3.1; *P* = 0.019).

**Conclusions:**

A pharmacogenomic-driven approach to clopidogrel prescribing has the potential to impact significantly on clinical management and outcomes in individuals of Bangladeshi and Pakistani ancestry.

Pharmacogenomics (PGx) explores how genetic variants contribute to interindividual variation in medication response.

Cytochrome P450 family 2 subfamily C member 19 (CYP2C19) is a hepatic enzyme crucial to the 2-stage sequential oxidation of clopidogrel (inactive prodrug) to the active metabolite.^1^ The *CYP2C19* gene that codes for the enzyme is highly polymorphic. Three key single nucleotide polymorphisms (SNPs) are referred to as ∗2, ∗3, and ∗17.^2^ The ∗2 (c.681G>A) and ∗3 (c.636G>A) SNPs both results in an early stop codon and, therefore, a truncated and nonfunctional protein (loss of function [LOF]).^3^ The ∗17 SNP (c.-806C>T) is a transition in the promoter region that increases enzyme expression and activity, thereby conferring gain of function leading to increase active metabolite.^3^ Pharmacokinetic studies have demonstrated lower active metabolite concentrations leading to decreased platelet response to clopidogrel in poor metabolizers (PMs) and intermediate metabolizers (IMs); the inverse is true of rapid and ultrarapid metabolizers, in a dose-dependent fashion.^4^^,^^5^ PMs and IMs have been linked with higher risk of secondary cardiovascular events on clopidogrel, while some studies have suggested rapid or ultrarapid metabolizers may have a higher risk of bleeding.^4^^,^^6^ Although there are known to be substantial trans ancestry differences in the prevalence of validated PGx SNPs ∗2, ∗3, and ∗17, many ethnic subgroups have not been investigated to date in resources with clinical outcome data.

Clopidogrel is an P2Y12-inhibiting antiplatelet medication licensed to treat acute coronary syndrome (ACS), stroke, and peripheral vascular disease.^7^ Although the European Cardiology Society and national guidelines have advocated the use of non–CYP2C19-dependent P2Y12 antagonist (ticagrelor and prasugrel) over clopidogrel in ACS owing to clinical trials showing superior efficacy, clopidogrel remains widely used.^8^^,^^9^ This may be due to concerns about the higher bleed risk, lower tolerability, and increased cost of ticagrelor and prasugrel as compared with clopidogrel. Clopidogrel is also the only antiplatelet agent to have a Class I indication in patients with stable coronary artery disease undergoing stent implantation and is recommended in those with a contraindication to ticagrelor or prasugrel or those taking an oral anticoagulant.^9^

Regulatory bodies and PGx consortia have given disparate guidance. The U.S. Food and Drug Administration recommends considering an alternate drug in PMs, while the European Medicines Agency merely discourages the co-use of CYP2C19 inhibiting drugs with clopidogrel.^7^^,^^10^ The Clinical Pharmacogenetics Implementation Consortium (CPIC) recommends an alternate therapy in IMs or PMs generally, but classifies PMs and those who have percutaneous coronary intervention (PCI) as populations at higher risk of treatment failure.^3^ The Dutch Pharmacogenetics Working Group recommends an alternate drug for PMs in those undergoing PCI and an alternate drug or increased dose of clopidogrel for IMs undergoing PCI.^11^ There is currently no routine implementation of *CYP2C19* testing in clinical care in the United Kingdom’s (UK) National Health Service (NHS), but this is likely to change as the National Institute for Health and Care Excellence recommends testing in the context of ischemic stroke.^12^

South Asian populations represents almost 25% of the world’s population and 9% of the United Kingdom.^13^^,^^14^ The UK–South Asian ancestry population is disproportionately affected by cardiometabolic disease and multimorbidity and suffer from a shortened life expectancy as compared with regional averages.^15^^,^^16^ Some of this can be attributed to disproportionate rates of socioeconomic deprivation.^13^ In addition, this population has been underrepresented in both clinical trial cohorts and genetics study cohorts historically. The objectives of this study were to assess *CYP2C19* genotypes in a British–South Asian ancestry cohort and to correlate inferred metabolizer phenotypes with recurrent myocardial infarction (MI) events in participants prescribed clopidogrel.

## Methods

### The Genes & Health cohort

The Genes & Health (G&H) resource was accessed after approval by the G&H Access Review Committee. G&H is a UK cohort study, including those of Bangladeshi and Pakistani ancestry.^13^ G&H operates under ethical approval, 14/LO/1240, from London South East NRES Committee of the Health Research Authority, dated 16 September 2014. The methods of data collection for the G&H resource have been previously described.^13^ In summary, >44,000 volunteers were recruited, donated saliva for DNA extraction, and gave consent to link to their electronic health records. Participants were genotyped on the Illumina GSAMD-24v3-0-EA chip, and imputation was undertaken using the TOPMED-r2 data set.^17^ The Genome Research Consortium human build 38 was used.

### Genotype/Imputation quality control

The ∗2 SNP was imputed, and the imputation quality was very high as assessed by imputation quality metric (imputation quality score) score (0.99). The ∗3 allele and ∗17 allele were genotyped. imputation quality score, minor allele frequency (MAF), Hardy-Weinberg equilibrium (HWE), and missingness for these 3 SNPs are shown in Supplemental Table 1. There was not substantial missingness. The population was not in HWE for the ∗2 and ∗17 alleles, likely due to previously reported relatedness (random mating is an assumption of HWE).^13^ However, the 3 SNPs used did not deviate from HWE in the subpopulation studied for clinical outcomes (those that had an MI and were treated with clopidogrel) (Supplemental Table 1).

### Characterization of *CYP2C19* genotype, diplotype, and inferred metabolizer phenotype in G&H cohort

Prevalence of the well-validated PGx *CYP2C19*∗2, *CYP2C19*∗3 and *CYP2C19*∗17 SNPs influencing CYP2C19 enzymatic function were ascertained. SNPs were extracted from the data set using PLINK 2.0.^18^^,^^19^ The ∗2 allele was defined as c.681G>A, rs4244285 (chr10:94781859). The ∗3 allele was defined as c.636G>A, rs4986893 (chr10:94780653). The ∗17 allele was defined as (c.-806C>T), rs12248560 (chr10:94761900).

Subsequent analysis was done using Rstudio.^20^ Any participant with 1 LOF SNP (either ∗2 or ∗3) was designated as an intermediate CYP2C19 metabolizer. Any participant with 2 LOF SNPs was characterized as a PM. Any participant with 1 ∗17 allele (in absence of a ∗2 or ∗3 allele) was designated a rapid metabolizer, and those with 2 ∗17 alleles were designated as ultrarapid metabolizers. The prevalence of these genotypes, diplotypes, and corresponding phenotypes were then compared with published population prevalence data provided by CPIC and with those represented in major recent randomized control trials (RCTs) evaluating PGx implementation.

### Linking CYP2C19 predicted phenotypes with recurrent myocardial infarction in participants who had an acute MI and were prescribed clopidogrel

Curated data sets from G&H were used for clinical phenotypes including acute MI (International Classification of Diseases [ICD]-10 code I21), subsequent MI (ICD-10 I22), Diabetes (DM) (E10; type 1 diabetes mellitus, E11; type 2 diabetes mellitus, E13; other specified diabetes mellitus, E14; unspecified diabetes mellitus), dyslipidemia (ICD-10 code E78), obesity (ICD-10 code E66), chronic kidney disease (ICD-10 code N18), and hypertension (ICD-10 code I10). These phenotypes were defined using ICD-10 codes, SNOMED codes, and Office of Population Censuses and Surveys codes from linkage with electronic health records, including Barts Health, NHS digital, Bradford teaching hospitals, and primary care clinical commissioning groups (CCGs). Participating CCGs included Barking, Havering and Redbridge, Tower Hamlets, Waltham Forest, and Newham.

The methodology used to generate these curated phenotypes is based on UK Biobank methodology and described in prior publications; the code is available on the G&H website.^21^, ^22^, ^23^ ICD-10 codes were identified in Barts Health NHS trust secondary care data, Bradford Teaching Hospitals NHS Trust, and NHS Digital hospital episode and mortality statistics. Primary care and secondary care SNOMED codes were then mapped to the ICD-10 code lists to capture the first recording of the code (1:1 mapping).

Medication data were obtained by linkage with primary care data. We limited our analyses to participants who had medication data available from participating CCGs (84.4% of the initial cohort). Participants diagnosed with an acute MI who had been prescribed 75 mg clopidogrel in primary care were assessed for recurrent MI events.

Smoking status was defined in primary care records by using SNOMED codes to distinguish never-smokers from those who had ever smoked (all codes are listed in Supplemental Table 2). Participants were classed as ever having smoked if they had a code in any of the smoking or ex-smoking categories and never having smoked if they did not have any codes associated with smoking or ex-smoking and had a code of never or currently not smoking. PCI with stent insertion was defined by ICD 10 code Z955, “presence of coronary angioplasty implant and graft” in Barts Health NHS Trust data.

### Statistical methods

Fisher’s Exact Test was used to compare baseline characteristics of participants who had a recurrent MI with those who did not, in the index acute MI population. T-test was used to compare means of continuous variables.

This was a cross-sectional analysis. Multivariable logistic regression was performed to look for association of *CYP2C19* diplotypes with recurrent MI in those who had been prescribed clopidogrel by the GP in the secondary prevention dose (75 mg). Four levels were used for the CYP2C19 inferred metabolizer type variable: PM, IM, and ultrarapid metabolizers, with normal and rapid metabolizers as the reference group. Sex, age at enrollment, and known cardiovascular disease comorbidities (diabetes mellitus, hypertension, dyslipidemia, obesity, chronic kidney disease, having ever smoked) were included as covariates. As the published literature suggests higher risk of in-stent thrombosis in PMs or IMs on clopidogrel, and a lower percentage of those who had stents were prescribed clopidogrel, PCI with stent insertion was also included as a covariate.^24^ There were 14 participants in the acute MI group, which included 1 participant in the recurrent MI group, for whom no PCI data were available. These participants were removed for logistic regression incorporating PCI data. The participant in the recurrent MI group who was excluded from this analysis due to the missing data was a normal CYP2C19 metabolizer. The 39 participants with recurrent MIs who had been prescribed clopidogrel were unrelated as assessed by the G&H curated KING analysis.^25^ Multicollinearity was assessed using the variance inflation factor of the car package in Rstudio.^26^

Sensitivity analysis was undertaken using the principal component analysis inferred ethnicity curated by G&H. 20 G&H curated principal components were included as covariates in a second logistic regression to control for population stratification. The G&H team have prior published data underpinning these variables, which they have made available to all researchers.^17^ Sensitivity analysis was also undertaken reclassing ∗2/∗17 and ∗3/∗17 diplotypes from intermediate to normal metabolizers.

A further analysis was undertaken in which *CYP2C19* LOF diplotypes were parametrized to a continuous variable where the unit was LOF alleles, 0 for none, 1 for one (IM), 2 for two (PM). Multivariable logistic regression analysis was then performed as above, controlled for age, sex, cardiovascular disease risk factors (smoking, diabetes mellitus, hypertension, obesity, dyslipidemia, chronic kidney disease), percutaneous coronary intervention, and 20 principal components. The 11 ultrarapid metabolizers were excluded from this analysis as outliers. This was undertaken to elucidate the impact of LOF alleles more clearly in absence of the ultrarapid diplotype which is an outlier in the trend of interaction between metabolizer type and clinical outcome.

## Results

### Characterization of *CYP2C19* genotypes in G&H cohort

The ∗2 SNP was very common in the G&H cohort (N = 44,396), with 56% of the population having at least 1 copy present. The ∗3 allele was less common, with 1.2% having at least 1 copy. In addition, 27.6% of the cohort had at least 1 copy of the ∗17 increased function allele.

#### *CYP2C19* diplotypes and inferred metabolizer phenotypes

**Figure 1:**
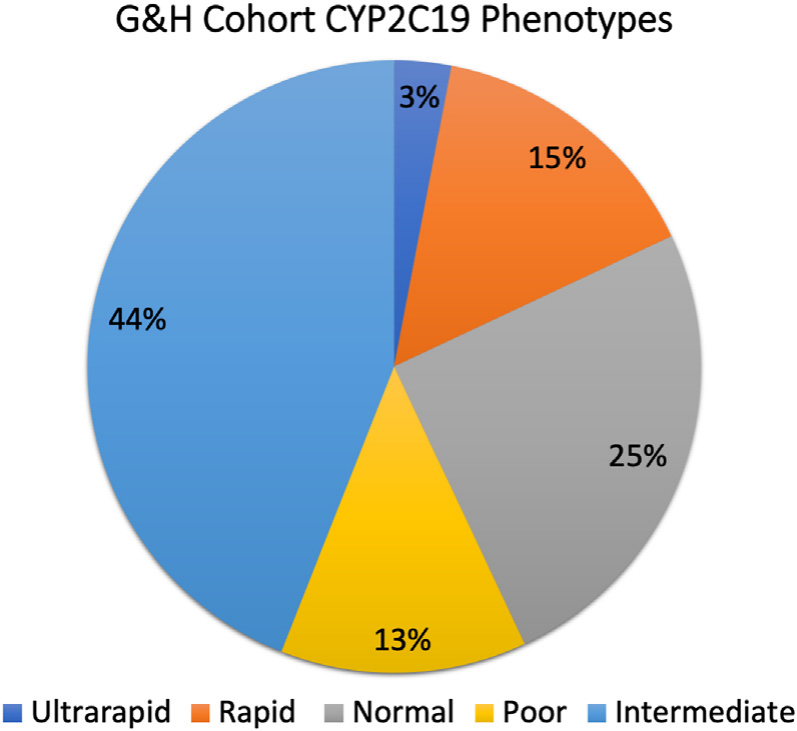
**Inferred CYP2C19 Metabolizer Phenotypes in G&H Cohort Population** CYP2C19 = cytochrome P450 2C19; G&H = Genes & Health.

PMs or IMs, carrying at least 1 LOF allele (∗2 or ∗3), comprised 57% of the cohort. Thirteen percent of participants were PMs, with 2 LOF alleles, while 44% were IMs, with 1 LOF allele (Figure 1). Furthermore, 2.7% of participants were ultrarapid metabolizers, homozygous for the ∗17 allele. Figure 1 illustrates that normal CYP2C19 metabolizers then represent only 25% of this population. This is a concern because it diverges from representation of SNP prevalence in largely European ancestry landmark clinical trials on PGx. Table 1 compares this cohort metabolizer status with expected in European and Central/South Asian populations and those reported in major recent PGx clinical trial cohorts assessing efficacy and safety of precision genomic-guided clopidogrel therapy.^27^, ^28^, ^29^, ^30^, ^31^

**Table 1:** Comparison With Biogeographic and Trial Cohorts^27^, ^28^, ^29^, ^30^, ^31^

Phenotype	G&H Cohort	CPIC Central/South Asian	CPIC European	TAILOR PCI Trial	POPular Genetics Trial
Rapid or ultrarapid	18%	21%	32%	a	a
Normal	25%	30%	40%	a	67%
Poor	13%	8%	2%	a	3%
Intermediate	44%	41%	26%	a	29%
Poor or intermediate	**57%**	**49%**	**29%**	**35%**	**31%**

The TAILOR PCI and POPular Genetics Trials were the 2 major randomized controlled trials to assess a genomic-guided approach to antiplatelet prescribing in ischemic heart disease.^30^^,^^31^ The Clinical Pharmacogenetics International Consortium (CPIC) considers a large number of ∗alleles, while we only considered only the 3 most-validated variants for clinical impact (∗2 and ∗3 together account for 99% of all LOF in Asian populations). Each row value is rounded to the nearest whole number. The **bold** values are the sum of all participants with a ∗2 or ∗3 LOF allele. G&H cohort N = 44,396. CPIC *CYP2C19* allele frequency estimates are based on Systematic Review of PubMed Indexed Publications; N = 7,100 for Central/South Asian and N = 71,782 for European.^3^ The TAILOR-PCI trial included 5,302 participants and the POPular Genetics Trial genotyped 1,242 participants.^30^^,^^31^

a Not specified.

#### Prevalence of clopidogrel prescriptions in the acute MI cohort

**Figure 2:**
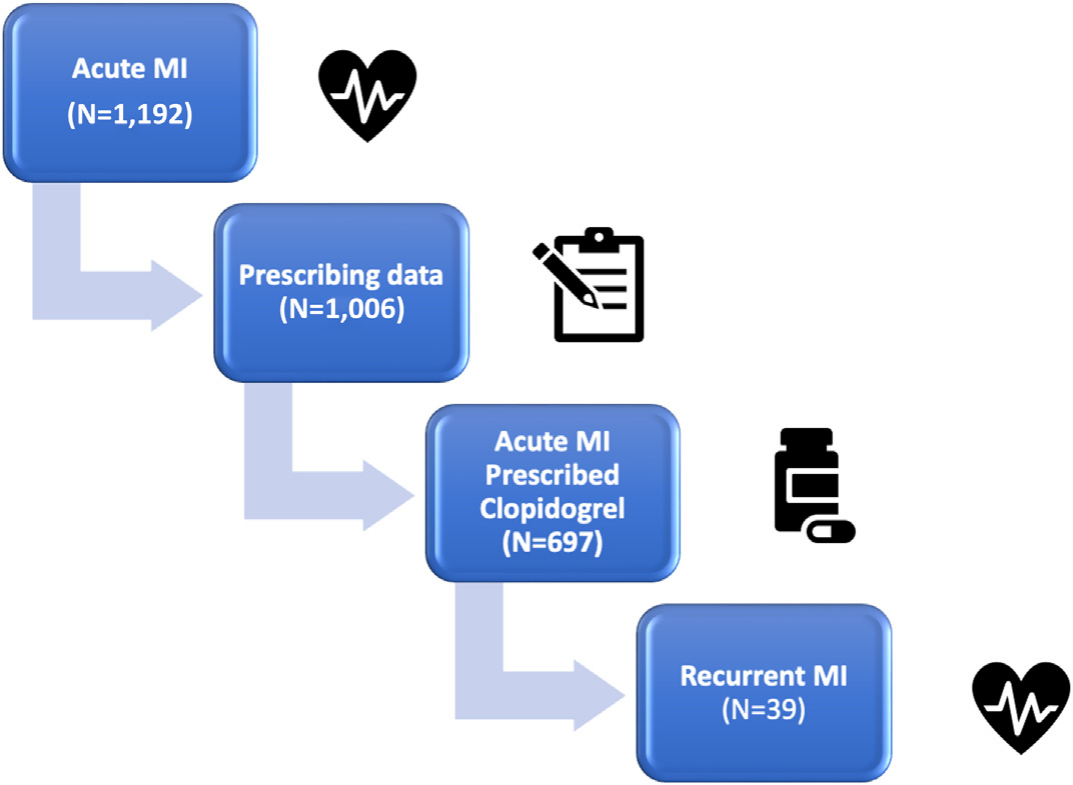
**Linking Medication Data With Clinical Outcomes** Primary care prescribing data was available for 84.4% of G&H participants diagnosed with an acute myocardial infarction (MI). 69% of these participants were prescribed clopidogrel. 5.6% of participants prescribed clopidogrel had a recurrent MI.

Medication data were available for those participants with linked primary care records (participating CCGs), which represented 84.4% of the acute MI cohort. We only included participants with prescribing data in our analysis. As antiplatelet choice post-MI is led by tertiary centers there is not expected to be any bias based on CCG linkage. The percentage of those who had an acute MI who were prescribed clopidogrel was 69.3% (Figure 2). For the subgroup who had a stent inserted, it was lower at 44.2%. Of those 697 participants who had an acute MI and were prescribed clopidogrel by their GP, 39 of them had recurrent MIs (5.6%) (Figure 2). Characteristics of the cohort with acute MI are noted in Table 2. Those with recurrent MIs were older and more likely to have PCI.

**Table 2:** Comparison of Cardiovascular Risk Factors and Outcomes in the Genes & Health Cohort Who Had Acute MI and Were Prescribed Clopidogrel

	Acute MI & Prescribed Clopidogrel (n = 697)	No Recurrent MI (n = 658)	Recurrent MI (n = 39)	*P* Value
Male	80% (556)	79% (520)	92% (36)	0.062
Diabetes	71% (493)	70% (460)	85% (33)	0.068
Hypertension	91% (633)	90% (595)	97% (38)	0.25
Dyslipidemia	90% (625)	89% (587)	97% (38)	0.17
Obesity	46% (218)	31% (206)	31% (12)	0.98
Chronic kidney disease	38% (267)	38% (247)	51% (20)	0.092
Smoker	90% (624)	90% (589)	90% (35)	1.00
Percutaneous coronary intervention	60% (410)	59% (380)	79% (30)	0.016^a^
Poor metabolizer	13% (90)	12% (81)	23% (9)	0.079
Intermediate metabolizer	43% (301)	43% (286)	38% (15)	0.62
Ultrarapid metabolizer	2% (11)	1% (8)	8% (3)	0.020^a^
Age at enrollment, y	61 ± 12	60 ± 12	67 ± 9	<0.001^a^

MI = myocardial infarction.

Values are % (n) or mean ± SD.

a *P <* 0.05.

#### Linking CYP2C19 phenotypes with clinical outcomes: recurrent MI in cohort prescribed clopidogrel

Multivariable logistic regression controlling for age, sex, cardiovascular comorbidities, smoking, and stent insertion showed a significant relationship between poor CYP2C19 metabolizers (OR: 3.1, 95% CI: 1.2-8.1, *P* = 0.019) and ultrarapid CYP2C19 metabolizers (OR: 10, 95% CI: 1.9-47, *P* = 0.003), and recurrent MI. IM status was not significantly associated with recurrent MI (*P* = 0.36) (Table 3). Reclassification of ∗2/∗17 or ∗3/∗17 from intermediate to normal metabolizers for sensitivity analysis did not change results. Apart from *CYP2C19* diplotype, increased age at recruitment was the only other factor found to be significantly associated with a recurrent MI event in the cohort prescribed clopidogrel (Table 3). Diagnosis of obesity, hypertension, dyslipidemia, diabetes mellitus, chronic kidney disease, or ever-smoking status were not significantly independently associated with recurrent MI risk in the cohort prescribed clopidogrel (though prevalence of all known risk factors was high). Multicollinearity was not found to be present. Sensitivity analyses to correct for the genetically determined subancestry group (British-Bangladeshi and British-Pakistani) did not alter the findings. Twenty principal components curated by G&H were controlled for to ensure our results were not biased by population stratification (Table 3). Table 4 shows the number of patients in the acute MI and recurrent MI cohort stratified by metabolizer status. There is a decrease in risk of recurrent MI on a gradient from PM to rapid metabolizer. There was an unexpected rise in risk in the ultrarapid metabolizer group, but there were only 11 participants in this group, as compared with 90-301 participants in the other metabolizer groups.

**Table 3:** Recurrent MI in Cohort Prescribed Clopidogrel

Metabolizer Phenotype	Risk of Recurrence of MI (OR)	95% CI	*P* Value
Poor CYP2C19 metabolizer (n = 9)	3.12	1.18-8.10	0.019^a^
	3.66	1.31-10.27	0.012^a^
Intermediate metabolizer (n = 15)	1.47	0.65-3.40	0.36
	1.78	0.75-4.46	0.20
Ultra-rapid metabolizer (n = 3)	10.49	1.94-47.51	0.003^a^
	19.77	3.17-110.73	0.001^a^
Increased age at recruitment	1.04 (per year)	1.01-1.08	0.009^a^
	1.05 (per year)	1.02-1.09	0.005^a^

Multivariable logistic regression analysis was used and controlled for age, sex, cardiovascular disease risk factors (smoking, diabetes mellitus, hypertension, obesity, dyslipidemia, chronic kidney disease) and percutaneous coronary intervention. The bottom values in each row are the principal component adjusted analysis results.

a *P <* 0.05.

**Table 4:** Ischemic Events in those Prescribed Clopidogrel Stratified by CYP2C19 Metabolizer Type (N = 697)

	CYP2C19 Metabolizer Status of the G&H Cohort Prescribed Clopidogrel					Total
	Poor Metabolizer	Intermediate Metabolizer	Normal Metabolizer	Rapid Metabolizer	Ultrarapid Metabolizer	
Acute MI	90	301	199	96	11	697
Recurrent MI	9	15	9	3	3	39
Percentage	**10.0%**	**5.0%**	**4.5%**	**3.1%**	**27.3%**	**5.6%**

Values are n or %. 90 participants were poor metabolizers (2 LOF alleles), 301 participants were intermediate metabolizers (1 LOF allele), 199 participants were normal metabolizers (no LOF or GOF alleles), and 96 participants were rapid metabolizers (1 GOF allele). There were very few participants in the ultrarapid metabolizer group (2 GOF alleles), N = 11. The percentage of participants in each metabolizer group to experience a recurrent MI is shown in **bold**.

As the primary interest for clinical implementation is *CYP2C19* LOF variants contributing to clopidogrel resistance, the 11 ultrarapid metabolizers were excluded and LOF diplotypes changed to a continuous variable where the unit was LOF allele (0, 1, or 2). This was undertaken to elucidate the impact of LOF alleles more clearly in absence of the ultrarapid diplotype which is an outlier in the trend of interaction between metabolizer type and clinical outcome (Table 4). The results show an OR of 1.95 (95% CI: 1.16-3.29 [*P* = 0.011]) per LOF allele (Table 5).

**Table 5:** Risk of Recurrent MI in Cohort Prescribed Clopidogrel

*CYP2C19* LOF Relationship With Recurrent MI Risk in Logistic Regression			
Risk Factor	Risk of Recurrence of MI, OR	95% CI	*P* Value
*CYP2C19* LOF allele	1.95	1.16-3.29	0.011^a^
Increased age at recruitment	1.05 (per year)	1.01-1.09	0.010^a^

Multivariable logistic regression analysis was used and controlled for age, sex, cardiovascular disease risk factors (smoking, diabetes mellitus, hypertension, obesity, dyslipidemia, chronic kidney disease) and percutaneous coronary intervention, as well as 20 principal components. CYP2C19 LOF diplotypes were parametrized to a continuous variable where the unit was LOF alleles, 0 for none, 1 for one (intermediate metabolizer), 2 for two (poor metabolizer). Ultrarapid metabolizers were excluded.

a *P <* 0.05.

## Discussion

People of diverse ancestry have different population prevalence of PGx SNPs, leading to different response to the same therapeutic agent between populations. A prospective PGx approach to prescribing, in which genotype is checked prior to medication prescription, is not standard of care at present. The case for implementation therefore must be proved as an improvement on current standards. Thus, the assumption is that we are at equipoise at baseline without implementation of PGx. This may be a flawed assumption as burden of prescribing without prospective genotype is not equally bourn by diverse ethnic groups. One of the underlying causes is that medications have historically been trialed in Caucasian ethnicity populations. The regulatory approval outcome studies of clopidogrel in ACS did not include representative participation from communities at higher risk of inefficacy based on genetic data^7^^,^^32^, ^33^, ^34^, ^35^, ^36^, ^37^, ^38^ (Supplemental Table 3).

This study demonstrates that the prevalence of poor and intermediate CYP2C19 metabolizers is higher in this British–South Asian ancestry cohort than previously known based on estimates of South Asian populations and substantially higher than has been shown in people of European ancestry. This is due to the high prevalence of the *CYP2C19* ∗2 allele in the G&H population. This work demonstrates the value of using real-world cohort study data to link CYP2C19 poor metabolizers with clopidogrel resistance. Although the ∗2 allele in this cohort may be particularly prevalent due to nonrandom mating, the prevalence of PMs and IMs is known to be very high in Asian populations generally.^29^ Marriage practices and kinship structure among South Asian populations may enrich certain variants in subgroups. Cardiovascular disease is known to be more prevalent in South Asian populations as compared with east Asian counterparts.

Given the increased cardiometabolic risk in this cohort, these participants therefore have both a higher risk of having an indication for clopidogrel and a higher risk of clopidogrel failure due to increased prevalence of *CYP2C19* LOF genotypes compared with counterparts of European ancestry. Our findings highlight the potential risk to diverse communities of licensure decisions based on evidence extrapolated from ethnically homogenous study populations. It is further problematic that large RCTs assessing potential benefit of genotype-guided prescribing of clopidogrel do not proportionately represent diverse global populations. It is interesting to note also that although the poor metabolizer diplotype was significantly associated with risk of recurrent MI in this cohort, the mere diagnosis of known cardiovascular disease risk factors (obesity, diabetes, dyslipidemia, hypertension, CKD, having ever smoker) were not. This is perhaps not surprising as the participants all have coronary artery disease already by definition but highlights the utility of *CYP2C19* genotyping as compared with clinical risk stratification.

British–South Asians in the G&H cohort are very likely to receive clopidogrel, with more than 2 in every 3 participants diagnosed with an acute MI in the G&H cohort receiving clopidogrel. International data suggest that this high prevalence of clopidogrel prescribing may be representative.^39^^,^^40^ A recent large Canadian cohort study of ACS patients who underwent PCI showed that 63.6% were prescribed clopidogrel (though clopidogrel use did decrease over time).^40^ One potential explanation for the continued prevalence of clopidogrel use is cost. In the United Kingdom, for example, the cost of 1 month of ticagrelor is 54.60 GBP as compared with clopidogrel, 1.24 GBP.^41^^,^^42^ Prasugrel is now off patent and therefore cheaper than ticagrelor, yet still more expensive than clopidogrel (cheapest available 10.14 GBP per month).^43^ In the United Kingdom, these cost differences are born by the NHS. In the global context, patients may have significant economic constraints relevant to decision-making if they are self-paying. These factors underscore the relevance of continued attention to clopidogrel PGx.

A pragmatic and often cited counter argument to genotype-guided antiplatelet prescribing is universal prescription of ticagrelor or prasugrel. However, the POPular genetics RCT trial has conclusively demonstrated that a genotype guided de-escalation of antiplatelet therapy, where poor or intermediate CYP2C19 metabolizers are given ticagrelor or prasugrel and others are given clopidogrel, is noninferior to universal ticagrelor/prasugrel prescription in terms of thrombotic events.^31^ Importantly, the risk of bleeding was significantly reduced with this approach (HR: 0.78; 95% CI: 0.61-0.98; *P* = 0.04).^31^ Therefore, if bleeding can be reduced and an equally efficacious medication used which costs less for those without CYP2C19 LOF alleles, genotyping seems likely to be cost-effective, particularly in the context of decreasing costs associated with genetic testing and/or a panel PGx approach. Indeed, real world health economic data published by the IGNITE -PGx group suggests that it would be; Limdi et al^44^ demonstrate that genotype guided escalation of therapy, using clopidogrel as the base case, is cost-effective compared with universal prescription of ticagrelor.

While these data convincingly demonstrate an increased risk of failed secondary prevention for CYP2C19 PMs prescribed clopidogrel, lack of signal for the IMs should not be taken as conclusive, given the limitations of the study that may mask such a signal (small sample size, lack of timeline data and compliance data, and lack of consideration of phenoconversion by drug-drug interaction). In the context of prior work showing that the lack of clopidogrel efficacy in carriers of LOF alleles is dose dependent, a finer tuned approach would likely be needed to detect a signal in intermediate metabolizers if one is indeed present. Likewise, the fact that only 60% of this cohort had PCI and only 44% of the PCI cohort overall was prescribed clopidogrel would be anticipated to lead to a weaker signal, as prior research suggests risk associated with in-stent thrombosis.

The data for risk to the small number of ultrarapid metabolizers in this cohort, taken in combination with prior pharmacokinetic studies and clinical trial data, suggest that they may be at higher risk of discontinuation due to adverse effects, but we cannot confirm or disprove this hypothesis with the data available. The risk of clopidogrel intolerance and discontinuation in ∗17/∗17 ultrarapid metabolizers has not been adequately assessed in clinical studies; future RCTs should genotype for ∗17 and assess for discontinuation rates and compliance in this subgroup as well as bleed-related adverse events. The number of patients who were ultrarapid metabolizers was very small (11 with acute MI and 3 with recurrent MI). Therefore, while showing an interesting real world data signal, these findings may be spurious and need to be validated in other studies. If validated it may capture a compliance risk that may not be equally represented in a controlled trial environment, which would have implications clinically (ie, support giving a non–CYP2C19-dependent antiplatelet to ultrarapid metabolizers). The large OR associated with ultrarapid metabolizer status, if due to discontinuation, would be consistent with pharmacokinetic and platelet aggregation evidence that suggests poor metabolizers may still have some active metabolite and thus some benefit from clopidogrel. In other words, that being a poor CYP2C19 metabolizer taking clopidogrel probably offers more protection than not taking clopidogrel or an alternative antiplatelet agent.^45^ All 3 of the ultrarapid metabolizer participants who had recurrent MIs had stents in situ, which would heighten the risk of clopidogrel discontinuation.

### Clinical implications

Given the prevalence of *CYP2C19* LOF alleles in the G&H cohort, this study supports genotyping in South Asian ancestry populations to guide antiplatelet prescription. It confirms that PMs have an elevated risk of failed secondary prevention of MI when receiving clopidogrel (Central Illustration). It combines this with new data showing higher than expected PM and IM prevalence in this British–South Asian cohort. Considering these results, caution should be used in extrapolating results from trials of European populations to diverse global populations, as conclusions may not be valid. Our results highlight risks inherent in prescribing medications to populations that vary widely from those including in studies used for licensure and postmarketing surveillance. When comparing safety and efficacy of clopidogrel to non–CYP2C19-dependent antiplatelet agents the ethnic composition of the cohort is likely to have an impact. Indeed, differences in data comparing efficacy of clopidogrel to ticagrelor may well be due to differences in ethnic representation. The PLATO trial, which included 6% Asian participants in the clopidogrel arm found a lower risk of the primary MACE endpoint in the ticagrelor group (in 9.8% of patients vs 11.7% at 12 months; HR: 0.84; 95% CI: 0.77-0.92; *P* < 0.001).^46^ The difference in risk of recurrent MI alone was significant but of a magnitude just over 1% (5.8% vs 6.9%, *P* = 0.005).^46^ A large Canadian cohort study found no difference in the efficacy of clopidogrel vs ticagrelor for secondary prevention but did not look at ethnic composition of the cohort.^40^ These results were reproduced by a large retrospective U.S.-based cohort study, which found no difference in the efficacy of clopidogrel vs ticagrelor for secondary prevention, but only included 1.2% Asians in the study.^39^ A Danish RCT published this year, which concurred that ticagrelor and clopidogrel did not have different efficacy in secondary prevention after PCI for ACS (cumulative incidence percentage 5.6% vs 6.0%; wIRR: 1.06, 95% CI: 0.92-1.22) did not publish or analyze ethnic makeup of the cohort. Given the biogeographic cohort, it seems likely to have been overwhelmingly European.^47^

**Central Illustration:**
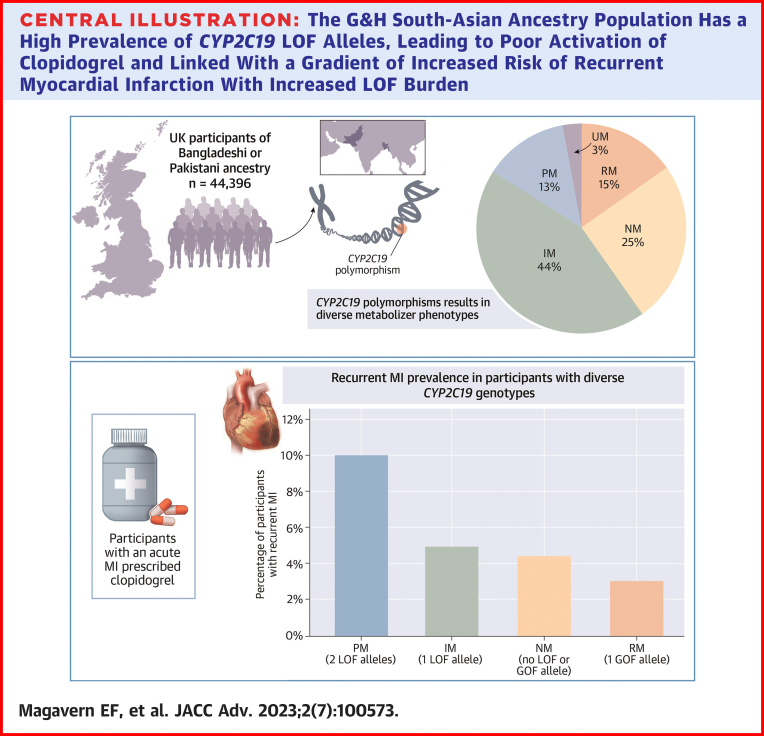
**The G&H South-Asian Ancestry Population Has a High Prevalence of *CYP2C19* LOF Alleles, Leading to Poor Activation of Clopidogrel and Linked With a Gradient of Increased Risk of Recurrent Myocardial Infarction With Increased LOF Burden** Due to the small number of ultra-rapid metabolizers present in the acute MI cohort, they were excluded from the graph showing recurrent MI risk. GOF = gain of function; IM = intermediate metabolizer; LOF = loss of function; MI = myocardial infarction; NM = normal metabolizer; PM = poor metabolizer; RM = rapid metabolizer; UM = ultra-rapid metabolizer.

Lack of action in terms of more diverse representation in trial cohorts and research cohorts risks perpetuating existing inequalities. More effort should be made to encourage publication of ethnic composition of research/trial cohorts especially in a setting where a PGx interaction between gene variants and a drug is probable and there is known transancestry differences in PGx variant prevalence.

### Study Limitations

Although the use of this real-world data has many advantages there are also some limitations. We did not have access to the dates of the index presentation and recurrent MI. Therefore, we cannot confirm that the recurrence of MI was during the time frame that the participants were prescribed clopidogrel. Furthermore, the risk of in-stent thrombosis after PCI is highest in the first 3 months poststent insertion, and this was not analyzed due to lack of timeline data. Limitation regarding timeline is mitigated by 2 factors: 1) built in temporality between index event and recurrent MI; and 2) the limited duration of dual antiplatelet therapy post-MI (usually 1 year).

Cause specific mortality data may also have refined our model, as a composite endpoint of recurrent MI or cardiovascular death could be considered, but these data were not available.

Furthermore, we did not consider coprescriptions that may cause drug-drug interactions or phenoconversion (ie, CYP2C19 inhibiting medication taken by a normal metabolizer which may convert them to an IM). This was due to our lack of timeline data and relatively small cohort. Comorbidities may also cause phenoconversion, for example diabetes is known to be associated with decreased CYP2C19 function.^48^

We did not have data to assess adverse events which may lead to clopidogrel discontinuation, for example significant gastrointestinal or intracranial bleed. However, the lack of the above data would be expected to mask any existing association between genotype and outcomes, meaning that our analysis would underrepresent rather than overrepresent a signal.

## Conclusions

This study shows high rates of genetically determined impaired clopidogrel activation in a large South Asian ancestry population with high risk of cardiovascular disease. Poor CYP2C19 activation of clopidogrel was correlated with an increased risk of recurrent MI. This “real-world” data show that clopidogrel is frequently prescribed and this appears to be particularly detrimental in individuals of South Asian ancestry due to the high proportion of PMs. Therefore, a pharmacogenomic-driven approach to antiplatelet prescribing has the potential to improve clinical management of MI and outcomes in individuals of Bangladeshi and Pakistani ancestry. High-quality health economic studies have shown that this approach is affordable.

In absence of a PGx approach, prescribing non–CYP2C19-dependent antiplatelet agents is likely to have a disproportionate benefit in this British-Bangladeshi and British-Pakistani population.

This study illustrates how socioeconomic deprivation, ethnic differences in pharmacogenes, and poor representation in research studies can intersect to compound ill health in an already disadvantaged subpopulation. Further analysis of such effects in clinical medicine are needed.

PERSPECTIVES **COMPETENTCY IN PATIENT CARE:** This work supports routine implementation of pharmacogenomic testing prior to prescribing clopidogrel for ACS. It links high prevalence of LOF genetic polymorphisms in the *CYP2C19* gene, which encodes the enzyme that converts clopidogrel to its active metabolite, with increased risk of re-current MI in a South Asian population. Furthermore, the study illustrates how systemic underrepresentation of this ancestral group in therapeutics trials has obscured the intersection of risks impacting this community. Pharmacogenomic testing prior to prescribing clopidogrel can improve health inequality and patient care.

**TRANSLATIONAL OUTLOOK:** To implement PGx equitably on a national and internationally scale, engagement work must take place so that prescribers and patients can build communication and data management pathways that are fit for purpose.

## Funding support and author disclosures

This work is part of the portfolio of research at the National Institute for Health Research (NIHR) Barts Biomedical Research Centre. Genes & Health is core-funded by Wellcome (WT102627, WT210561), the Medical Research Council (UK) (M009017), Higher Education Funding Council for England Catalyst, Barts Charity (845/1796), Health Data Research UK (for London substantive site), and research delivery support from the NHS National Institute for Health Research Clinical Research Network (North Thames). Genes & Health is/has recently been funded by Alnylam Pharmaceuticals, Genomics PLC; and a Life Sciences Industry Consortium of Bristol-Myers Squibb Company, GlaxoSmithKline Research and Development Limited, Maze Therapeutics Inc, Merck Sharp & Dohme LLC, Novo Nordisk A/S, Pfizer Inc, Takeda Development Centre Americas Inc. Dr Magavern is funded by Barts Charity. All other authors have reported that they have no relationships relevant to the contents of this paper to disclose.
