# Supplementary material for: CYP2C19 Genotype Prevalence and Association With Recurrent Myocardial Infarction in British–South Asians Treated With Clopidogrel
Source: JACC Adv. 2023 Aug 21;2(7):100573. doi: 10.1016/j.jacadv.2023.100573 (PMC10550831; doi:10.1016/j.jacadv.2023.100573)

**Supplemental table 1: Genotype and imputation metrics**

| **SNP** | ***2** | ***3** | ***17** |
| --- | --- | --- | --- |
| **Mutation** | c.681G>A | c.636G>A | c.-806C>T |
| **Chromosome location** | chr10:94781859 | chr10:94780653 | chr10:94761900 |
| **Rs** | rs4244285 | rs4986893 | rs12248560 |
| **MAF** | 0.34 | 0.006 | 0.15 |
| **HWE p-value**  **(N=44,396)** | <0.001 | 0.17 | <0.001 |
| **MAF (N=697)** | 0.34 | 0.007 | 0.14 |
| **HWE p-value MI analysis**  **(N=697)** | 0.45 | 1 | 0.53 |
| **Proportion of sample missing (N=44,396)** | 0.00045 | 0.001509 | 0 |
| **INFO Score** | 0.99 |  |  |

**Supplemental table 2: SNOMED codes used to ascertain smoking history**

| Never or current nonsmoker codes | Current or ex-smoker codes including those associated with smoking harm and cessation advice |
| --- | --- |
| 266919005 | 8517006 |
| 160601007 | 65568007 |
| 160618006 | 77176002 |
| 8392000 | 229819007 |
|  | 266922007 |
|  | 266923002 |
|  | 266924008 |
|  | 225323000 |
|  | 266918002 |
|  | 160617001 |
|  | 225324006 |
|  | 160616005 |
|  | 266925009 |
|  | 160606002 |
|  | 160603005 |
|  | 266920004 |
|  | 228487000 |
|  | 281018007 |
|  | 134406006 |
|  | 160612007 |
|  | 2.031910e+14 |
|  | 3.659810e+08 |
|  | 3.948710e+08 |
|  | 394871007 |
|  | 1.572871e+15 |
|  | 1.964110e+14 |
|  | 7.830110e+14 |
|  | 1.857950e+08 |
|  | 1.857960e+08 |
|  | 1.857920e+08 |
|  | 1.857990e+08 |
|  | 185799001 |
|  | 1.85795e+08 |
|  | 5.27151e+14 |
|  | 1.377110e+14 |
|  | 1.084381e+15 |
|  | 4.010680e+08 |
|  | 7.650010e+08 |
|  | 1.572871e+15 |
|  | 3.152320e+08 |
|  | 8.71661e+14 |
|  | 8.71641e+14 |
|  | 3.95700e+08 |
|  | 5.05281e+14 |
|  | 7.67641e+14 |
|  | 401068004 |

**Supplemental Table 3**: Studies supporting clopidogrel licensure as listed in the European Medicines Agency summary of product characteristics(7,32–38) . The only study with a substantial Asian population was focused on stroke rather than myocardial ischemia and undertaken in East Asia.

**Studies supporting Clopidogrel licensure (EMA)**


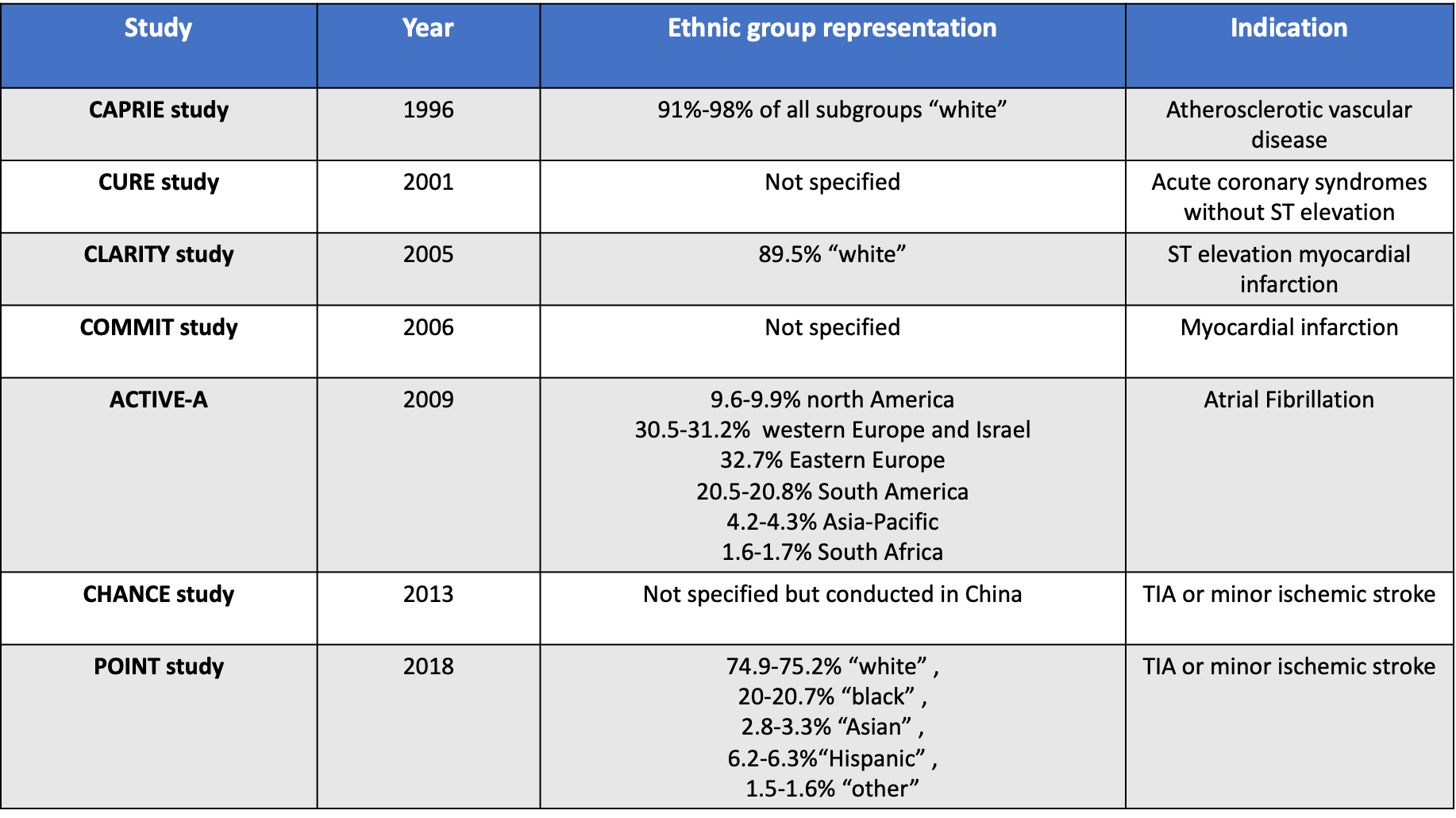

Supplement: Supplementary data [file mmc1.docx]
